# Tumor-derived extracellular vesicle proteins as new biomarkers and targets in precision oncology

**DOI:** 10.1007/s00109-024-02452-6

**Published:** 2024-05-30

**Authors:** Haiyan Liao, Cheng Zhang, Fen Wang, Feng Jin, Qiqi Zhao, Xinying Wang, Shubin Wang, Jing Gao

**Affiliations:** 1 Department of Oncology, Shenzhen Key Laboratory of Gastrointestinal Cancer Translational Research, Cancer Institute, Peking University Shenzhen Hospital, Shenzhen-Peking University-Hong Kong University of Science and Technology Medical Center, Shenzhen, China; 2 Key Laboratory of Carcinogenesis and Translational Research (Ministry of Education/Beijing), Department of Gastrointestinal Oncology, Peking University Cancer Hospital and Institute, Beijing, China; 3 Chi Biotech Co., Ltd., Shenzhen, China

**Keywords:** Extracellular vesicle proteins, Biomarkers, Tumor diagnosis, Tumor microenvironment, Precision medicine

## Abstract

Extracellular vesicles (EVs) are important carriers of signaling molecules, such as nucleic acids, proteins, and lipids, and have become a focus of increasing interest due to their numerous physiological and pathological functions. For a long time, most studies on EV components focused on noncoding RNAs; however, in recent years, extracellular vesicle proteins (EVPs) have been found to play important roles in diagnosis, treatment, and drug resistance and thus have been considered favorable biomarkers and therapeutic targets for various tumors. In this review, we describe the general protocols of research on EVPs and summarize their multifaceted roles in precision medicine applications, including cancer diagnosis, dynamic monitoring of therapeutic efficacy, drug resistance research, tumor microenvironment interaction research, and anticancer drug delivery.

## Introduction

Extracellular vesicles (EVs), also known as exosomes, microvesicles, microparticles, ectosomes, oncosomes, apoptotic bodies, and many other names [1], are cell-secreted vesicles of different sizes and intracellular origins and are of increasing interest to scientists due to their numerous functions in physiology and pathology [2, 3]. EVs contain diverse biomolecules, including proteins, lipids, metabolites, mitochondrial DNA [4], mRNA [5], other noncoding RNAs, and regulatory RNA [6], that are important for homeostasis; they act by transferring nucleic acids and specific repertoires of proteins and lipids [2, 7]. Of these, extracellular vesicle protein (EVP) components can be categorized into two major types. One type is EVPs found in various cell types. They are involved in vesicle formation and secretion and commonly used as exosome markers; these EVPs include membrane transport and fusion-related proteins (e.g., GTPase), heat shock proteins (e.g., HSP70), members of the four-transmembrane protein superfamily (e.g., CD63), ESCRT complex-related proteins (e.g., Tsg101), and integrins. The other type is EVPs closely related to cell specificity, such as tetraspanins (e.g., CD37 and CD53 in leukocytes), ERBB2 in breast cancer, CD45 in immune cells, and major histocompatibility complex class (MHC) and II [1, 8]. EVs perform many functions in fundamental physiological processes, such as neuronal communication [9], immune response [10], organ development [11], reproductive performance [12], cancer progression [13], cardiovascular disease [14], and inflammation [15]. The role of EVs in cancer research is increasingly appreciated because cancer cells secrete at least tenfold more EVs than normal cells [16, 17] and can be stably detected in various kinds of body fluids, such as blood, urine, saliva, and bronchoalveolar fluid. In addition, by transferring oncogenic proteins and nucleic acids, tumor-derived EVs interact with the microenvironment and are involved in tumorigenesis, growth, progression, and drug resistance [18–20].

**Fig. 1:**
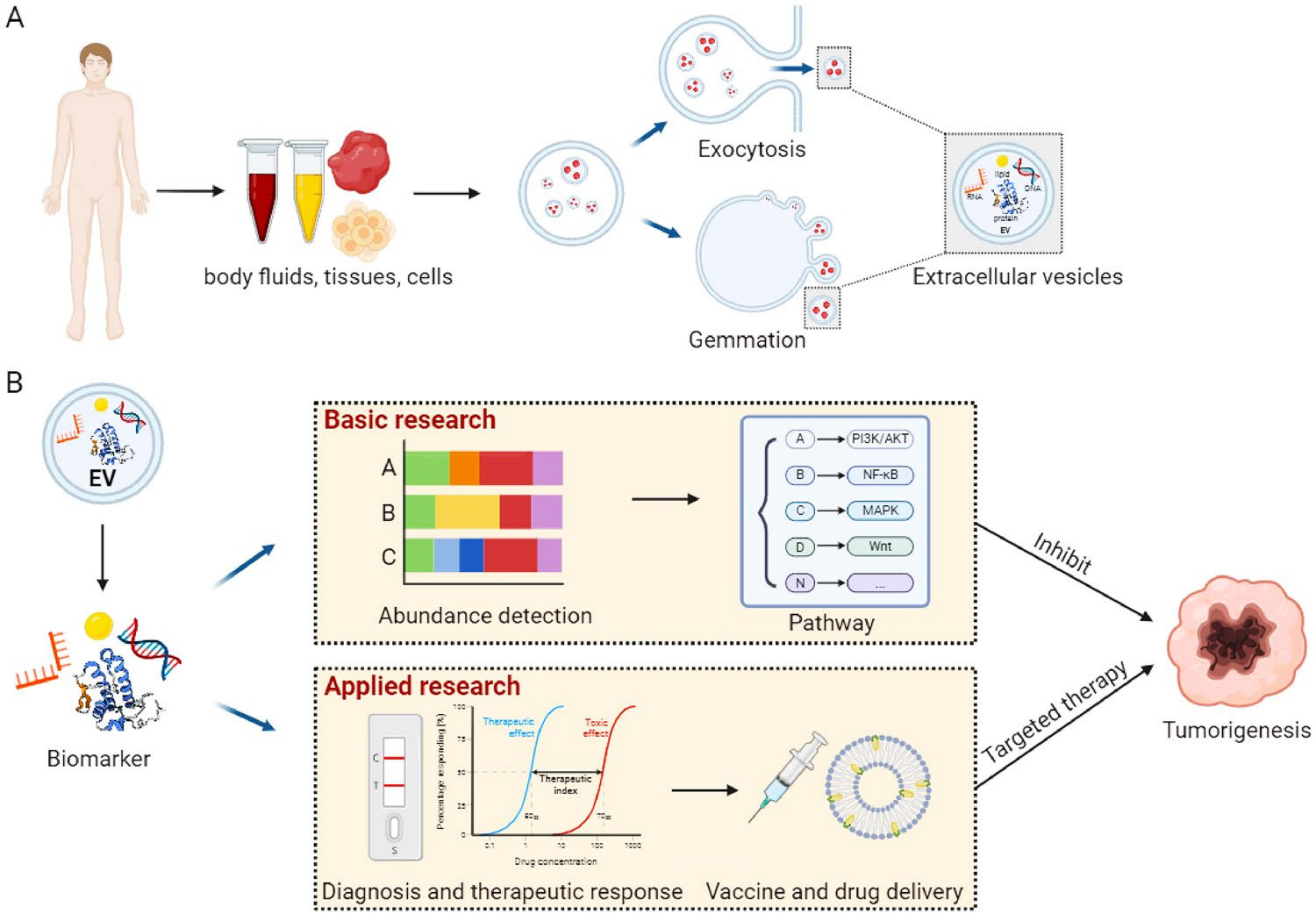
Precision biomedical applications of extracellular vesicles. **A** Extracellular vesicles obtained from body fluids, tissues, and cells can be derived from exocytosis and gemmation. **B** Precision biomedical applications of EV intracellular components. Basic research: detecting biomarker abundance and further targeting anti-tumor immunity through signaling pathways. Applied research: biomarkers are used as disease diagnosis and therapeutic response, further developing vaccines and drug delivery to target tumor

The incidence of cancer is growing rapidly worldwide, with an estimation of more than 18 million new cases and more than 9 million deaths per year [21–23]. EVPs of tumor cells are increasingly drawing attention for tumor monitoring because of their diverse characteristics [24, 25] and applications, ranging from tumor diagnosis [26], prognostication [27], dynamic monitoring, drug resistance, and precise targeted drug delivery [28] (Fig. 1). In this review, we discuss the developments in the isolation and characterization strategies of EVPs and highlight their functions in the tumor microenvironment and recent applications in precision oncology, such as tumor diagnosis, treatment monitoring, and drug delivery.

## Isolation and identification of EVPs

**Fig. 2:**
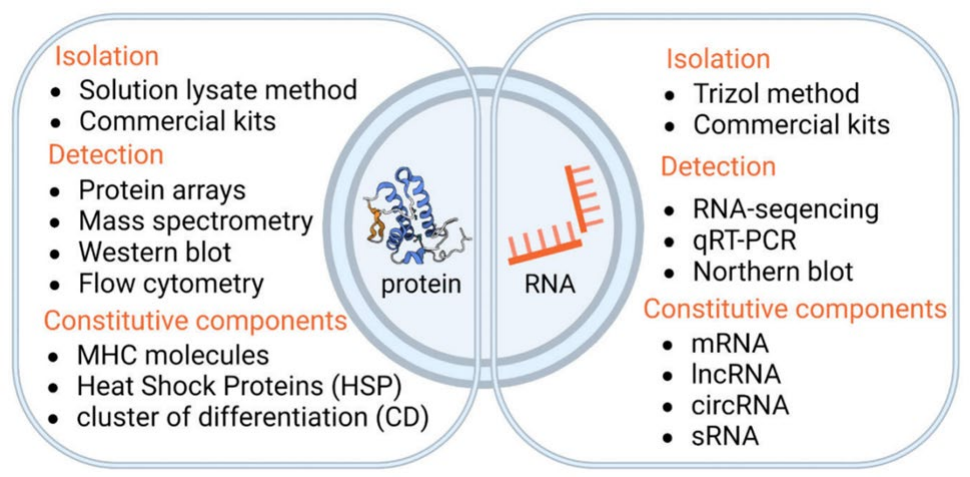
Differences in the isolation, detection and constitutive components of extracellular vesicle proteins and RNAs. A comparison of standard and emerging techniques for the isolation and detection of extracellular vesicles and RNAs and a general overview of constitutive components

Most of the studies on EVs focus on their proteins and RNA; however, the research methods are different due to the various purposes and characteristics of RNA and protein (Fig. 2). Existing studies of EVs usually involve (1) isolation, purification, and characterization of EVs and (2) identification of the composition of EVs and analysis of their data profile. The small size, low density, and contaminants that normally coexist with EVs make their isolation and purification difficult and not reproducible. Therefore, efficient enrichment of EVs is currently a major issue and is crucial for downstream analysis. Separation methods should be carefully chosen for different purposes and applications relying on either the physical or biochemical properties. The traditional methods include differential ultracentrifugation, size-exclusion-based chromatography, density gradients, polymer precipitation, and immunoaffinity capture techniques [29]. In addition, recent studies have developed several novel techniques for EV separation and enrichment. Lactoferrin-conjugated 2,2-bis-(hydroxymethyl)-propionic acid dendrimer-modified magnetic nanoparticles (LF-bis-MPA-MNPs), chimeric nanocomposites, were used for simple and sensitive EV isolation and cancer detection from human urine samples [30]. A surface plasmon ultrasensitive method based on polymer dots and AuNPs was used for detecting EVs in pancreatic cancer with high sensitivity [31]. Microfluidic systems with multiple confinement structures can be used for capturing and quantifying circulating EVs from small sample volumes and applied to clinical studies [32]. For the characterization of isolated EVs, electron microscopy, nanoparticle tracking analysis, Western blotting, and flow cytometry are commonly used. These methods have accelerated the study of extracellular vesicles.

Proteins in EVs have a high potential for application in tumor diagnosis due to their stable nature, which can provide rich information, and the extraction of proteins from EVs has become a new hotspot. Traditionally, EVPs are generally extracted in RIPA buffer supplemented with protease inhibitors [33] or obtained by digestion in trypsin. There are differences in the EVPs content separated by different methods and buffers, and it was recommended in MISEV2018 that the properties and concentration of the reagents be clearly indicated [1]. EV lysis with RIPA buffer resulted in the highest detectable amount of proteins when compared against other buffers, such as Tris-SDS lysis, Tris-Triton lysis, and Guanidium chloride lysis, suggesting that RIPA buffer is an optimal lysis buffer for EV proteomics analysis based on mass spectrometry [34]. Trypsin has been also widely used in the literature for protein extraction, and various colorimetric assays, Western blotting, and mass spectrometry have been applied for measurement after isolation of EVs from cells or body fluids [35, 36]. Nevertheless, EV proteins that belong to intravesicular cargo are thought to be resistant to trypsin treatment because trypsin cannot penetrate the vesicular membrane [37]. A strategy based on using membrane impermeant biotin coupled to mass spectrometry analysis has been reported for the analysis of EV surface protein expression, which would avoid compromising EVP integrity by Proteinase K [38]. Several methods have been developed to identify EVPs after the extraction of EVs from body fluids or cells, conventionally including mass spectrometry and enzyme-linked immunosorbent assay (ELISA) methods combined with quantitative reverse transcription polymerase chain reaction (qPCR), Western blotting, or flow cytometry. In addition, several new techniques, such as microfluidic chips [39] and adaptive dynamic artificial poly-ligand targeting (ADAPT) [33], are now available.

With the rapid development of proteomic technologies and analytical methods for EVPs, targeted analysis of the membrane proteins on the surface is of great scientific importance. Tian and colleagues reported a rapid method based on a laboratory-built high-sensitivity flow cytometer (HSFCM) for protein profiling and size detection of EVs in colorectal cancer (CRC) patients. The researchers found that HSFCM allows immunofluorescence staining and enables protein analysis of individual EVs and quantitative counting of different EV subsets [40]. Ko et al. proposed an antibody-based immuno-sequencing method called seiSEQ, which enabled highly sensitive detection of specific proteins at the single EV level by isolating and coding individual EVs using droplet microfluidics, followed by sequencing of barcodes/antibody-DNA to determine protein composition [41]. Moreover, with the development of omics technologies, several databases for studying EV have become available. ExoCarta (http://www.exocarta.org) is a commonly used database on EVs that contains data from more than 286 EV studies, including data on 41,860 proteins, providing data support for conducting EVP studies. All separation, extraction, and analytical techniques have helped us recognize EV and EVP and have accelerated EV-related research.

## Functions of EVPs in the tumor microenvironment

The malignancy of a tumor depends not only on the tumor cells themselves but also on the interaction between the tumor and its microenvironment (TME) [42]. The TME consists of various cell types, including stromal cells (e.g., fibroblasts, mesenchymal stromal cells, pericytes, and adipocytes) and immune cells (e.g., T and B lymphocytes, natural killer (NK) cells, and tumor-associated macrophages (TAMs)), all of which can be embedded in the extracellular matrix (ECM) [43, 44]. In recent years, numerous studies have proven that tumor-derived EVPs play important roles in shaping the tumor microenvironment by activating tumorigenesis, remodelling the ECM, re-educating stromal cells, or regulating immune cell interactions, thereby facilitating cancer development [45–47].

Tumor-derived EVs contain a range of tumor-specific antigens and ligands that interact with corresponding receptors on immune cells. Immune checkpoint molecules, including PD-1/PD-L1 and CTLA-4, have been reported to be present in EVs isolated from various cancer types, and these EVPs have been implicated in the regulation of the immune microenvironment and response to immunotherapy. Exosomal PD-L1 from tumor cells systemically suppress antitumor immunity by directly binding to PD-1-expressing CD8+ T cells, and the level of plasma exosomal PD-L1 may predict the clinical effectiveness of anti-PD-1 therapy [48]. Poggio et al. also proved that cancer cells can secrete their PD-L1 protein into exosomes and hence suppress T-cell activation [49]. In addition, other tumor-derived EVPs associated with the microenvironment have been reported. A study indicated that HSP72 expressed at the surface of tumor-derived exosomes, acting as a ligand that binds to TLR2 on myeloid-derived suppressor cells (MDSCs), is responsible for activating human MDSCs and triggering their suppressive function via the activation of Stat3 [50]. Similarly, tumor exosomal PGE2 and TGF-β can also induce the accumulation of MDSCs [51]. Fas ligand expressed on tumor-derived exosomes was proven to induce apoptosis of activated T lymphocytes [52]. Moreover, CDCP1 was found to be a novel tumor-associated antigen in EVs from irradiated tumor cells, which can trigger antitumor immunity against primary tumors and experimental lung metastases by enhancing the infiltration of CD8+ and CD4+ T cells [53].

The survival rate of cancer patients treated with immune checkpoint therapy (ICT) and vitamin E (VitE) was significantly increased, and increasing evidence has shown that EVs are involved in the regulation of the tumor immune microenvironment and influence the efficacy of antitumor therapy. VitE enters dendritic cells (DCs) through the SCARB1 receptor and restores the function of tumor-associated DCs by directly binding and inhibiting the protein tyrosine phosphatase SHP1. Enhanced cross-presentation of tumor antigens on DCs and DC-derived extracellular vesicles (DC-EVs) triggers systemic antigen-specific T-cell antitumor immunity [54]. HSPC111-rich EVs secreted by colorectal cancer cells can transform quiescent fibroblasts into tumor-associated fibroblasts, which reprogram their lipid metabolism and promote the synthesis and secretion of acetyl-CoA. Acetyl-CoA promotes the synthesis and secretion of the chemokine CXCL5 through the acetylation of histone H3K27, which binds to the chemokine receptor CXCR2 on the surface of CR cells, promoting high expression of HSPC111 in CR cells and its secretion in EVs. Moreover, acetyl-CoA promotes the proliferation and migration of colorectal cancer cells, triggers epithelial-mesenchymal transition, and induces the metastasis of colorectal cancer cells to the liver [55]. Within the tumor microenvironment, EVs can help cancer cells proliferate and spread, as well as help them evade the immune system, and they can act as carriers of molecules that can kill cancer cells and reactivate the immune system.

## Application of EVPs in tumor diagnosis

Currently, mounting evidence supports a role for tumor-derived EVPs in diagnosing or predicting the state of cancer (Table 1), as EVPs have a higher concentration in cancer patients than in nontumor individuals and contain much cancer-associated biological information. In addition, EVPs are considered to have a higher sensitivity and specificity than proteins directly detected in body fluids [56–58]. Plasma exosomal ephrin type-A receptor 2 (EphA2) levels in pancreatic cancer (PC) patients who received neoadjuvant chemotherapy or chemoradiotherapy were significantly lower before and after treatment in patients with curable PC but not in those without PC, suggesting that EphA2 could be used for monitoring the curative effect in PC [59]. CPN1, IGHV2-26, ITIH3, CLU, and DNAJB11 derived from plasma exosomes were also found to be associated with PC progression and may represent alternative biomarkers for diagnostic [60, 61]. In another study, Glypican-1 (GPC-1)-positive EVs were proven to be a diagnostic indicator of early PC [62]. Reports have found that CD317, epidermal growth factor receptor (EGFR) in plasma EVs, and human leucine rich alpha-2-glycoprotein 1 (LRG1) in urinary EVs are reliable biomarkers for diagnosing non-small cell lung cancer (NSCLC) [63, 64]. CD91 in EVs, as a lung adenocarcinoma (ADC)-specific antigen, can help identify patients with tumors [65]. In both small cell lung cancer (SCLC) and NSCLC, plasma-derived MUC5B, SELL, and APOH have been shown to be potential biomarkers for the diagnosis of brain and liver metastasis. Latent membrane protein 1 (LMP1) could be detected in EVs from nasopharyngeal carcinoma (NPC) cell lines [66]. Additionally, LMP1 and BARF1 could be detected in EVs in the serum and saliva from teenagers and adults with nasopharyngeal carcinoma [67]. Exosomal CYPA, which associated with Epstein-Barr virus (EVB), has been verified as potential diagnostic biomarker for EVP-positive NPC [68]. Additionally, analysis of the EVPs expression level in the blood sample could distinguish hepatocellular carcinoma (HCC) patients from healthy individuals and revealed significantly elevated galectin-3-binding protein (G3BP) and polymeric immunoglobulin receptor (PIGR) in EVPs of hepatocellular carcinoma (HCC) patients [69]. Tripartite motif-containing 3 (TRIM3) and gastrokine 1 (GKN1) were found to be downregulated in plasma exosomes in gastric cancer (GC) compared with those in healthy controls, so these factors are thought to be novel biomarkers for GC diagnosis and therapeutic targets [70, 71]. Heat shock protein 60 (HSP60), serpin peptidase inhibitor clade A member 1 (SERPINA1) and fibrinogen (PLG) in the plasma EVs of patients is a promising candidate for CR diagnosis [72, 73]. Different cancer types, including BC, CRC, PC, lung cancers, and mesothelioma, could be distinguished by specific combinations of patients’ plasma-derived EVPs, and these cancer-specific EVPs can be used as liquid biopsy tools to help diagnose patients with cancers of unknown primary tumor origin and to guide the treatment of these patients [74].

**Table 1:** Extracellular vesicle proteins (EVPs) act as diagnostic biomarkers for various cancers

**Disease**	**Biomarkers**	**Biofluid**	**Year**	**References**
**Lung cancer**	CD91	Plasma	2014	Ueda et al. [65]
	CD317, EGFR		2015	Jakobsen et al. [63]
	Versican		2023	Chang et al. [78]
	FGB, FGG, VWF		2023	Luo et al. [79]
	MUC5B, APOH, CD81, CCT5		2023	Li et al. [80]
	LRG1	Urine	2011	Li et al. [64]
**Nasopharyngeal carcinoma**	LMP1	Plasma	2006	Keryer et al. [66]
	CYPA		2019	Liu et al. [68]
	BARF1	Saliva	2007	Houali et al. [67]
**Pancreatic cancer**	EphA2	Plasma	2017	Liang et al. [59]
	CPN1/IGHV2-26/ITIH3/CLU		2023	Marin et al. [60]
	DNAJB11		2023	Liu et al. [61]
	GPC-1		2024	Li et al. [62]
**Hepatocellular carcinoma**	G3BP, PIGR	Plasma	2017	Arbelaiz et al. [69]
**Cholangiocarcinoma**	CRP/FIBRINOGEN/FRIL/PIGR	Plasma	2023	Lapitz et al. [81]
**Gastric cancer**	TRIM3	Plasma	2018	Fu et al. [70]
	GKN1		2018	Yoon et al. [71]
**Colorectal cancer**	HSP60	Plasma	2015	Campanella et al. [72]
	CD147		2018	Tian et al. [40]
	SERPINA1, PLG		2023	Li et al. [73]
**Ovarian carcinoma**	TGF-beta, MAGE3/6	Plasma	2013	Szajnik et al. [75]

Numerous studies have confirmed that proteomic analysis could distinguish cancer-derived EVs from normal cell-derived EVs, and EVPs vary with the tissue origin of cancer cells. Szajnik et al. found from 22 patients’ plasma samples that TGF-beta and MAGE3/6 proteins present on exosomes can distinguish ovarian carcinoma from benign tumors or nontumor tissues, and the levels of these tumor-associated proteins correlate with patients’ responses to therapy [75]. A study performed on the proteomic profile of EVs secreted by sixty cell lines from the National Cancer Institute (NCI-60) found that only 213 of 6071 identified EV cargo proteins were common to all cell lines. The researchers further confirmed that most EVPs clustered based on cancer type; that is, each cancer cell type secretes EVs with a unique proteomic cargo, indicating the potential value of EVPs as circulating biomarkers for identifying and diagnosing different cancer types [76]. Hoshino et al. performed a comprehensive proteomic analysis of EVPs from 426 human samples (including tissue explants, plasma, and other bodily fluids) and identified a panel of tumor-associated EVPs (e.g., VCAN, TNC, and THBS2) that could be used as biomarkers for early-stage cancer detection with more than 90% sensitivity and specificity. The specific combinations of these EV proteins could be used to diagnose tumors of unknown primary origin [74]. Hinestrosa et al. developed a blood-based EVP detection method and identified a panel of 13 EVPs that allowed the detection of early-stage pancreatic, ovarian, and bladder cancers with an average sensitivity of 71.2% and specificity of 99.5% [77]. By comparing plasma EVs from 37 CRC patients and 32 healthy individuals using the HSFCM platform, Tian et al. confirmed the high predictive value of CD147 expression in EVs for CRC diagnosis [40]. Therefore, tumor-derived EVPs could be used for early cancer detection and identifying tumors of unknown origin.

## Application of EVPs in predicting therapeutic effects

Currently, biomarkers that can predict therapeutic effects are urgently needed to rapidly screen for therapeutically superior options and assist in the clinical adjustment of treatment strategies through real-time monitoring. EVPs, as liquid biopsy tools, similar to CTCs and ctDNA, have been proven to have good efficacy, predictive value, and clinical application prospects in various tumors (Table 2). The results of a clinical trial (NCT02862470) in patients with recurrent thyroid cancer showed that although serum thyroglobulin was not detected in 3 patients with thyroid cancer, the levels of urinary exosomal thyroglobulin were consistently increased, indicating that urinary exosomal thyroglobulin protein is a potential marker for predicting thyroid cancer recurrence [82]. Lin and colleagues found that serum EVP concentration and protein characteristics could be used as prognostic predictors in colorectal liver metastasis (CRLM) patients in both discovery and validation cohorts, which included 56 and 154 CRLM patients, respectively [83]. In addition, the extracellular vesicle protein chemokine ligand 7 (CXCL7) can be used as a biomarker to predict early response in patients with CRLM undergoing chemotherapy [83]. A study established an EV^DX^ signature (defined as a weighted sum of the expression of the following 8 EV markers) by linear discriminant analysis of eight breast cancer-associated serum EVPs (CA15-3, CA125, CEA, HER2, EGFR, PSMA, EpCAM, and VEGF), which can distinguish metastatic breast cancer (MBC) patients from non-MBC patients and healthy donors with an overall accuracy of 91.1% [84]. More importantly, the EV^DX^ signature showed high accuracy in monitoring the MBC treatment response in the training (88.9%), validation (87.9%), and prospective (85.2%) cohorts and was associated with progression-free survival (PFS). Additionally, investigations using EVPs to predict the efficacy of immunotherapy and targeted therapies have been applied in cancer. It was reported that PD-L1 could be secreted extracellularly via EV or in the soluble form, except for its expression on the cell membrane [85]. Moreover, plasma exosomal PD-L1 is closely related to the response to immunotherapy, affecting the prognosis of patients. Furthermore, it has been confirmed in melanoma, GC, and NSCLC that patients with low baseline peripheral blood exo-PD-L1 levels usually have complete or partial responses to PD-1/PD-L1 inhibitor therapy [86–88]. Therefore, circulating exosomal PD-L1 is expected to be a new predictive marker for the efficacy of immunotherapy with PD-1 inhibitors. In conclusion, EVPs are increasingly being used to predict and monitor the response to treatment.

**Table 2:** Extracellular vesicle proteins (EVPs) act as treatment response biomarkers of various cancers

**Disease**	**Biomarkers**	**Biofluid**	**Year**	**References**
**Gastric cancer**	PD-L1	Plasma	2019	Fan et al. [85]
	HER2		2023	Li et al. [89]
**Pancancer**	PD-1	Plasma	2020	Zhang et al. [86]
			2021	Del et al. [87]
**Melanoma**	PD-1/PD-L1	Plasma	2022	Serratì et al. [88]
**Breast cancer**	CA15-3, CA125, CEA, HER2, EGFR, PSMA, EpCAM, VEGF	Plasma	2017	Tian et al. [84]
**Urothelial carcinoma**	FR-α, TLR 3, TRAIL, FASLG	Plasma	2022	Viktorsson et al. [90]
**Thyroid cancer**	Thyroglobulin	Urine	2020	Huang et al. [82]
**Colorectal cancer**	CXCL7	Plasma	2022	Lin et al. [83]
**Hepatocellular carcinoma**	GPX3/ACTR3, ARHGAP1	Plasma	2022	Shuen et al. [91]

## Application of EVPs in monitoring drug resistance

Primary or secondary drug resistance was considered the main cause of treatment failure. The secretion of EVs can act as mediators of signal transduction, altering the sensitivity of tumors to chemotherapeutic drugs and participating in tumor progression. Investigation of the drug resistance mechanism of EVs and their associated proteins can be helpful to improve the therapeutic effect of tumors. It has been shown that ovarian cancer cells excrete drug molecules directly by secreting EVs. EVs alter the sensitivity of target cells to chemotherapeutic drugs by transporting active proteins and affect the chemoresistance of ovarian cancer cells by modulating the tumor microenvironment [92]. EVs released from drug-resistant breast cancer cells are rich in EphA2 protein, which promotes tumor invasion and metastasis through the EphA2-Ephrin A1 reverse pathway [93], and the increase in EphA2 in EVs released from drug-resistant cell-derived cells may be an important mechanism of chemoresistance-induced breast cancer progression. EVs from gemcitabine-resistant cells in triple-negative breast cancer promote gemcitabine resistance in sensitive cells through the regulation of EGFR by the extracellular vesicle annexin A6 (ANXA6) protein [94]. Cisplatin-resistant NSCLC cells can be induced to secrete EVs in a hypoxic environment and transfer drug resistance to sensitive NSCLC cells via intercellular delivery of pyruvate kinase isozyme type M2 (PKM2) [95]. Exosomal Wnts from fibroblasts can induce dedifferentiation of tumor cells to promote chemoresistance in CRC, suggesting that interference with exosomal Wnt signaling contributes to greater chemoresistance [96]. The elevated levels of four membrane protein biomarkers in melanoma plasma EVs, melanoma chondroitin sulfate proteoglycan (MCSP), melanoma cell adhesion molecule (MCAM), low-affinity nerve growth factor receptors (LNGFR), and receptor tyrosine protein kinase and ErbB3 may predict resistance to B-Raf proto-oncogene serine/threonine kinase (BRAF) inhibitor therapy in melanoma patients [97]. Research to date strongly supports the pivotal role of EVs in tumor resistance.

## Application of EVPs in drug delivery

Compared with liposomes and viral vectors, EVs are characterized by high stability, good biocompatibility, low immunogenicity, and excellent biological barrier penetrability (such as the blood‒brain barrier and placental barrier). Therefore, EVs are considered natural nanomaterials that can be used as drug delivery vehicles with great prospects for targeted therapies [98, 99]. A study fused platelet EVs with photothermal-sensitive liposomes to obtain biomimetic nanocarriers, which not only retained their physiological functions, such as platelet-macrophage escape, tumor selective adhesion, and damaged blood vessel targeting, but also improved drug-carrying capacity [100]. Targeted drug delivery to tumor tissue exerted a synergistic therapeutic effect. Zhang et al. developed an engineered neutrophil-like EV-like nanovesicle that could precisely target tumor tissue for efficient antitumor effects [101]. Antibody-modified and tumor antigen peptide-stimulated EVs from dendritic cells were used as T-cell activators to build “bridges” between cancer cells and activated T cells, thereby enhancing the antitumor immune response of T cells [102]. Engineered macrophage EVs were modified with biodegradable nanodrug carriers for precise sonodynamic therapy of gliomas [103]. In summary, engineered EVs and their protein cargos are expected to be new tools for tumor treatment with minimal side effects.

## Conclusion and perspective

**Fig. 3:**
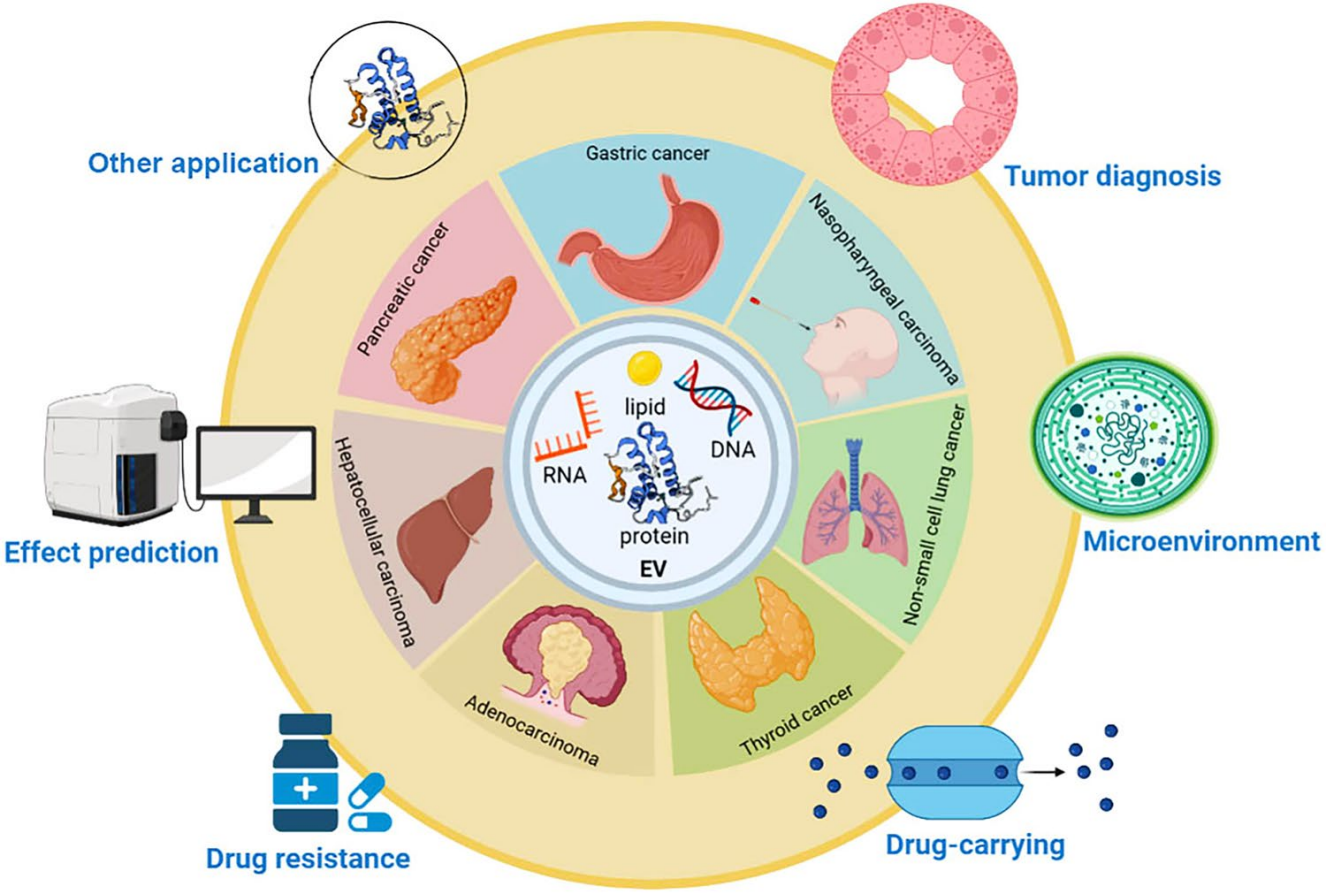
The application of extracellular vesicular proteins in cancer

EVPs, which play crucial roles in TME (tumor microenvironment) regulation and drug resistance, represent a new field as an effective therapeutic approach in cell-free medicine and are increasingly used in tumor diagnosis, therapeutic monitoring, and drug-carrying with amazing results based on the advantages of EV stability (Fig. 3). However, for precise clinical treatment, the source of EVs must be selected cautiously. For example, tumor-derived EVs exhibit remarkable targeting ability to tumor cells but also contain bioactive substances that promote tumor progression [104]. To further improve the targeting efficiency of EVs and reduce adverse effects, the integration of multifield technologies can be used to modify EVs so they can respond to specific external triggers (e.g., light, sound, heat, and magnetic fields) to specifically target lesions. Currently, drug loading of EVs can be realized by incubation, transfection, sonication, and electroporation to achieve clinical translation of the therapeutic effects of EVs in tumors.

Notably, although the current findings suggest the great potential of EVPs in precise diagnosis and treatment, there is still much work to be done before they can be used in clinical practice. There is a lack of simple, rapid, and standardized clinical assays for EVs and a lack of experience with clinical trials of EV-related contents. However, the development of a large number of EV-based formulations bodes well for the widespread clinical use of EVPs in the future. Tumor-associated EVPs could be used as biomarkers for early cancer detection, therapeutic response, and potentially for the diagnosis of primary unknown tumors. There is potential for the implementation of routine EVP-based screening in the clinical setting. In the future, exploring the important role of EVPs in vivo and their unique properties will facilitate the beneficial applications of EVs for patients and society.

## Data Availability

Not applicable.

## Abbreviations

EV: Extracellular vesicle
MV: Microvesicle
EVP: Extracellular vesicle protein
TME: Tumor microenvironment
ECM: Extracellular matrix
NK: Natural killer cell
TAM: Tumor-associated macrophage
DC: Dendritic cell
MDSC: Myeloid-derived suppressor cell
NSCLC: Non-small-cell lung cancer
HCC: Hepatocellular carcinoma
BC: Breast cancer
MBC: Metastatic breast cancer
PC: Pancreatic cancer
GC: Gastric cancer
CRC: Colorectal cancer
CRLM: Colorectal cancer liver metastases
